# Sleep as a reflexion of well-being in schizophrenia

**DOI:** 10.1192/j.eurpsy.2026.12148

**Published:** 2026-07-31

**Authors:** D. Njah, M. Lagha, K. Maagli, A. Zyed, W. Homri

**Affiliations:** Psychiatry c, Razi psychiatric hospital, Manouba, Tunisia

## Abstract

**Introduction:**

Sleep disorders affect between 30% and 80% of people with schizophrenia (Dule *et al.* TOPHJ. 2020;13(1):684‑91). These disturbances have been linked to an exacerbation of psychiatric symptoms, a decline in cognitive function and a substantial reduction in quality-of-life (QoL) scores across various domains. These findings emphasize the importance of treating sleep disorders specifically to enhance the overall well-being of patients with schizophrenia (Chen *et al.* Schizophr Res 2024;264:407‑15).

**Objectives:**

Our objective was to assess the impact of sleep quality on the QoL in patients with schizophrenia and schizoaffective disorder (SAD).

**Methods:**

This was a cross-sectional study including patients with schizophrenia and SAD, aged between 18 and 65 years, who were followed up at RAZI psychiatric Hospital in Tunisia. Sleep quality was assessed using the Arabic version of the Patient Sleep Quality Index (PSQI). Quality of life was assessed using the French version of the 12-item Short Form (SF-12) scale.

**Results:**

Fifty patients with schizophrenia and SAD were included in our study. The mean age was 42 ± 12 years, 60% were men, and 82% were single. The median global PSQI score was 11 [4; 13], ranging from 0 to 17. The median physical health score (PCS-12) was 42.23 [31.08; 52.72], while the mean mental health score (MCS-12) was 42.91 ± 11.12. Both PCS-12 and MCS-12 scores were below the general population average of 50. According to the PSQI scale, 24% of patients were categorized as good sleepers, while 76% were identified as poor sleepers. A significant association was observed between the global PSQI score and PCS-12, with lower PSQI scores corresponding to higher PCS-12 scores (β = -1.887, p = 0.001). The PSQI score accounted for 68.2% of the variance in PCS-12 (R² = 0.682). Additionally, a significant association was found between the global PSQI score and MCS-12 (p < 0.001). Simple linear regression indicated that higher PSQI scores were associated with lower MCS-12 scores (β = -1.835, p < 0.001), with the PSQI score explaining 69% of the variance in MCS-12 (R² = 0.69).

**Conclusions:**

Poor sleep quality significantly affects both physical and mental well-being of patients with schizophrenia or SAD. Therefore, systematic screening and management of sleep disturbances are crucial to optimize overall functioning and daily outcomes in this population.

**Disclosure of Interest:**

None Declared

